# The Effect of Hepatic Surgical Margins of Colorectal Liver Metastases on Prognosis: A Systematic Review and Meta-Analysis

**DOI:** 10.3390/jcm13247776

**Published:** 2024-12-19

**Authors:** Daniel Paramythiotis, Eleni Karlafti, Dimitrios Tsavdaris, Fani Apostolidou Kiouti, Anna-Bettina Haidich, Aristeidis Ioannidis, Stavros Panidis, Antonios Michalopoulos

**Affiliations:** 1 First Propaedeutic Surgery Department, University General Hospital of Thessaloniki AHEPA, Aristotle University of Thessaloniki, 54636 Thessaloniki, Greece; danosprx@auth.gr (D.P.); tsavdaris@auth.gr (D.T.); ariioann@yahoo.gr (A.I.); st.panidis@gmail.com (S.P.); amichal@auth.gr (A.M.); 2 Emergency Department, University General Hospital of Thessaloniki AHEPA, Aristotle University of Thessaloniki, 54636 Thessaloniki, Greece; 3 First Propaedeutic Department of Internal Medicine, University General Hospital of Thessaloniki AHEPA, Aristotle University of Thessaloniki, 54636 Thessaloniki, Greece; 4 Department of Hygiene, Social-Preventive Medicine and Medical Statistics, School of Medicine, Faculty of Health Sciences, Aristotle University of Thessaloniki, University Campus, 54124 Thessaloniki, Greecehaidich@auth.gr (A.-B.H.)

**Keywords:** colorectal liver metastasis, surgical margins, prognosis, systematic review, meta-analysis

## Abstract

**Introduction:** Colorectal cancer is the third most common malignancy, with around half of patients developing liver metastases. Hepatectomy is the preferred treatment, but its success depends on several factors, including surgical margins. Various surgical margins have been suggested to achieve optimal results. This systematic review and meta-analysis aim to explore the impact of negative surgical margins ranging from 1 to 10 mm, and >10 mm on survival, with the objective of identifying optimal surgical margins. **Methods:** A systematic literature search was conducted on the MEDLINE, Scopus, and Cochrane databases. The six included studies that examined the effect of surgical margins at the aforementioned distances on patient survival. Studies were assessed for risk of bias using the Quality in Prognosis Studies tool. Statistical analysis was performed using SPSS software. **Results:** The results of the meta-analysis revealed the superiority of wider surgical margins (>10) on overall survival compared to smaller margins (1–10 mm), as the HR was calculated to be 1.38 [1.10; 1.73]. Specifically, negative margins between 1 and 10 mm are linked to a 38% increased risk of mortality compared to margins larger than 10 mm. The low heterogeneity indicates consistent findings across studies, and the statistically significant hazard ratio underscores the importance of aiming for larger surgical margins to enhance patient outcomes. In the subgroup that included only studies in which patients received neoadjuvant therapy, the HR was 1.48 [1.06; 2.07], further emphasizing the importance of ensuring negative surgical margins in today’s era. **Conclusions:** In summary, this systematic review and meta-analysis highlights the impact of surgical margin width on the survival of patients with colorectal liver metastases, as well as the importance of margin optimization in surgical management strategies.

## 1. Introduction

Colorectal cancer (CRC) stands as the predominant malignancy within the gastrointestinal system, ranking as the third most prevalent malignant neoplasm within the general population [1]. The seriousness of this malignancy is perceived by its ranking as the second leading cause of cancer-related mortality [2]. Epidemiological observations suggest variable incidence rates contingent upon geographic locale and racial demographics, exhibiting a notable rise with advancing age [3,4]. Contributory factors include familial predisposition, a personal medical history characterized by polyps or adenomas, dietary habits, and inflammatory bowel conditions [5,6,7]. Approximately one-fifth of CRC patients present with distant metastatic spread [8]. Notably, the liver serves as a frequent site of metastasis due to its anatomical proximity and the distinctive circulatory dynamics inherent to the body’s vasculature, particularly implicating the portal vein system whereby the majority of colonic venous outflow converges [8,9].

The liver emerges as the predominant site of metastatic involvement in CRC, with nearly half of CRC patients eventually developing hepatic metastases [10,11]. This high incidence of hepatic metastases is accompanied by a burdened prognosis for affected individuals. Current therapeutic strategies for managing colorectal liver metastases (CRLM) include chemotherapy or multimodal approaches [12]. Nonetheless, hepatic resection remains the treatment of choice, yielding favorable outcomes characterized by 5-year overall survival (OS) rates ranging from 40% to 60% [13,14]. However, the success of hepatic resection is influenced by a multitude of factors, including both surgical technique and perioperative management [15]. Importantly, the adequacy of hepatic surgical margins is a crucial technical consideration due to its substantial impact on patient prognosis [16,17,18].

In the context of CRLM, surgical margin status refers to the extent of cancerous tissue excised during surgical resection. This status is categorized as negative (R0) or positive (R1) based on the absence or presence of tumor cells at the surgical margin, respectively [19,20]. Notably, surgical margin status is closely correlated with the prognosis of affected patients, while they are also associated with the recurrence of malignancy as well as with other distant metastases. R0 resection, characterized by the absence of tumor cells at the surgical margin, is linked with the most favorable long-term outcomes and a reduced risk of recurrence [19,20,21].

Over time, several ideal widths and extents of surgical margins have been proposed to optimize patient survival. The conclusions of various studies differ regarding the safest surgical margin, as many studies state that a surgical margin of >10 mm is required [22,23,24,25,26,27], while others argue that a margin exceeding 1 mm is sufficient, with no additional benefit observed at greater distances [28,29,30,31,32]. It is therefore considered necessary to investigate the optimal surgical margins that ensure the best patient outcomes.

The aim of this systematic review and meta-analysis is to analyse the effect of different negative liver thresholds (specifically, negative margins ranging from 1 to 10 mm versus margins greater than 10 mm) on overall patient survival in individuals undergoing resection for colorectal liver metastases. By elucidating the impact of surgical margins on survival, this study endeavors to inform surgical practices, facilitate judicious decision-making regarding surgical strategies and patient care, and potentially inspire quality enhancement initiatives aimed at refining surgical techniques.

## 2. Materials and Methods

### 2.1. Study Protocol and Guidelines

This systematic review and meta-analysis was conducted in accordance with the Preferred Reporting Items for Systematic Reviews and Meta-Analyses (PRISMA) guidelines [33]. This systematic review and meta-analysis is registered in the Open Science Framework (OSF), with the registration number: 10.17605/OSF.IO/TB5QE

### 2.2. Eligibility Criteria

The eligibility criteria for this systematic review and meta-analysis are outlined and were established using the PICO framework. Studies were considered for inclusion if they met the following criteria:

- Population: adult patients with colorectal liver metastases, either at the initial diagnosis or following liver resection.
- Intervention: surgical resection of liver metastases arising from colorectal carcinomas with clearly defined hepatic surgical margins.
- Comparison: evaluation of surgical margins categorized into two groups: margins ranging from 1 to 10 mm and margins greater than 10 mm.
- Outcome: provision of hazard ratios for overall survival comparing the two margin groups.

Studies were excluded if they were reviews, letters, comments, surveys, meta-analyses, pilot studies, conference abstracts, or case reports. Additionally, non-English language articles were excluded. Additionally, studies reporting Kaplan–Meier survival curves alone and not providing the appropriate data to calculate HRs were excluded. Only original research articles meeting the specified criteria were considered for inclusion in this review.

### 2.3. Information Sources, Search Strategy, and Selection Process

The search was executed within the online databases MEDLINE, Scopus, and Cochrane library, employing the search terms (colorectal OR colon) AND (metastasis OR metastases) AND (liver OR hepatic) AND resection AND (margin OR margins). No search filter was used and there was no time limit. The selection process involved two independent reviewers (D.T. and E.K.) who assessed the eligibility of studies for inclusion in this systematic review and meta-analysis based on the title, abstract, and full-text evaluation.

### 2.4. Data Collection Process and Data Items

Data extraction was performed independently by two reviewers (D.T. and E.K.). Any discrepancies between reviewers were resolved by involving a third reviewer (D.P.), who provided an additional review to ensure accuracy and consistency. The data were extracted into a standardized Excel form, without the assistance of any automation tools. The key information extracted from the reviewed studies included the main characteristics of each study, such as the author and year of publication, location, type of research, total subjects, recruitment period, median follow-up, as well as overall survival (OS) percentages and hazard ratios (HR) with 95% CI. The primary endpoint is the OS of the patients with 1–10 mm negative surgical margins as well as with >10 mm negative surgical margins. The efficacy effect of overall survival will be calculated using HR, as it is the most appropriate metric, given that it accounts for the time factor during the follow-up period.

### 2.5. Study Risk of Bias Assessment

Two reviewers (D.T. and E.K.) independently conducted the risk of bias assessment for each included study. Discrepancies between reviewers were resolved by involving a third reviewer (D.P.), who reviewed the conflicting assessments and provided a final judgment.

The risk of bias in the included studies was assessed using the QUIPS (Quality in Prognosis Studies) tool. This tool is an evaluation scale that uses six domains, and each domain is judged on a three-grade scale (low, moderate, or high risk of bias). The first category concerns the participants in each study to judge the risk of selection bias, while the next category concerns study attrition to investigate the likelihood that the relationship between the prognostic factor and outcome are different for completing and non-completing participants. The third area investigates the risk of measurement bias related to measuring the prognostic factor [34].

### 2.6. Statistical Analysis

The statistical analysis was performed using an RStudio integrated development environment (IDE) by two reviewers (F.A.K. and D.T.). Statistical analysis was performed using R statistical software v4.4.1, developed by The R Foundation for Statistical Computing, located in Vienna, Austria, specifically with the meta and metafor packages. The process began with preparing the data, which consisted of HRs and their corresponding 95% confidence intervals (CIs) from the six included studies. The data were organized into a data frame with columns for the study names, HRs, lower bounds of the CIs, and upper bounds of the CIs.

To facilitate the meta-analysis, hazard ratios were log-transformed to stabilize the variance and make the results more interpretable. The standard errors for these log-transformed hazard ratios were calculated using the following formula:

$$SE\;=\;\left(ln\left(upper\_ci\right)\;-\;ln\left(lower\_ci\right)\right)\;/\;\left(2\;*\;qnorm\left(0.975\right)\right)$$

where *qnorm*(0.975) represents the quantile function of the standard normal distribution at the 97.5th percentile.

The meta-analysis itself was conducted using the metagen function from the meta package in R. This function allowed for the specification of both fixed-effect and random-effects models. The analysis was performed with the following parameters: hazard ratios were specified as the measure of effect size, and results were backtransformed to hazard ratios from their log-transformed values. The comb.fixed parameter was set to TRUE to include the fixed-effect model, while the comb.random parameter was set to TRUE to include the random-effects model. The DerSimonian–Laird method was used for tau-squared estimation.

The results of the meta-analysis were summarized using the summary function, which provided an overview of the pooled hazard ratios along with their confidence intervals. To visually represent the data, a forest plot was generated using the forest function. This plot displayed the individual study hazard ratios and the overall pooled estimate, with a reference line at HR = 1 to denote no effect. The forest plot also included a prediction interval to account for variability among studies.

Additionally, a funnel plot was created using the funnel function to evaluate potential publication bias and the symmetry of the results. This plot provided a graphical representation of the effect sizes versus their standard errors, aiding in the assessment of the robustness of the meta-analysis findings.

## 3. Results

### 3.1. Study Selection

**Figure 1:**
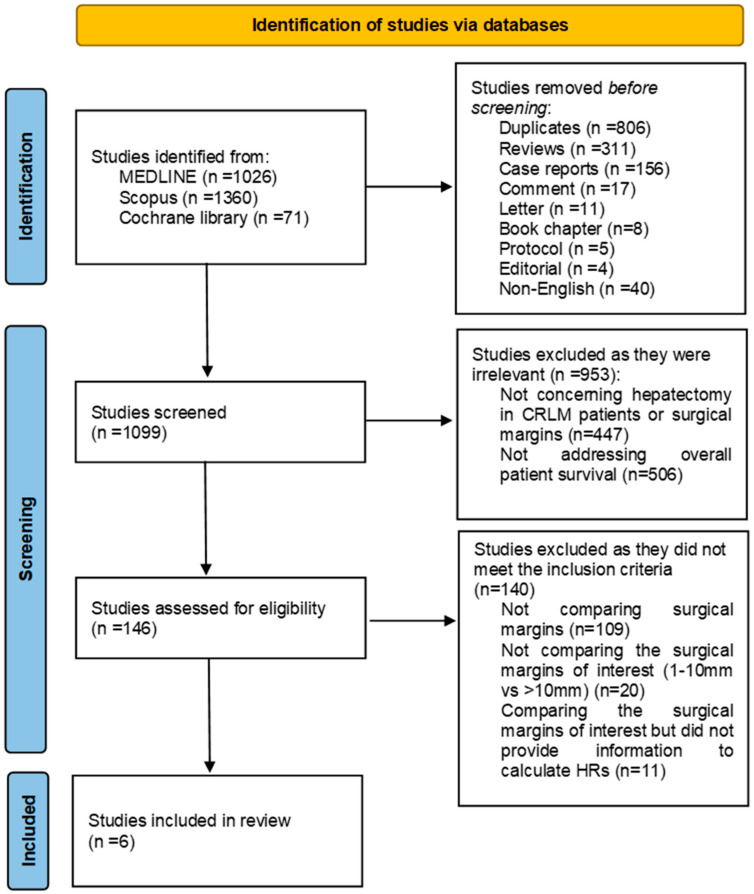
PRISMA 2020 flow chart.

Using the keywords (colorectal OR colon) AND (metastasis OR metastases) AND (liver OR hepatic) AND resection AND (margin OR margins) in MEDLINE, Scopus, and the Cochrane Library, 2457 results were found. After excluding duplicates, reviews, letters, comments, surveys, meta-analyses, pilot studies, conference abstracts, and case reports, 1099 studies were screened, with only 146 of them analyzing the prognosis of surgical margins in patients with CRLM. Of these 146 studies, only 6 studies met the inclusion criteria, i.e., examined the requested treatment groups (1–10 mm and >10 mm surgical margins). The selection process for the studies included in this systematic review and meta-analysis is depicted in a PRISMA 2020 flow chart (Figure 1).

### 3.2. Study Characteristics

Six studies were included in this systematic review and meta-analysis [35,36,37,38,39,40]. Three thousand three hundred ninety-two individuals participated in these studies, with a clear predominance of the male gender, and the age nearing the sixth decade of life. These are six observational retrospective studies, which took part in five countries. The recruitment period varies between the six studies, starting in 1950 and ending in 2019 in the most recent study. The median follow-up exceeds three years in all studies, with the exception of Fonseca et al. [39], which reports a median follow-up duration of 34 months, showing the reliability of the results. In the studies reporting the number of R1s [35,36,40], the prevalence of negative surgical margins becomes clear as 600 R0s versus 53 R1s are noted. Finally, regarding the substratification of negative margins in the treatment groups (1–10 mm and >10 mm surgical margins), the prevalence of surgical margins that extend from 1 to 10 mm becomes clear, noting 1764 patients as opposed to the 1056 patients in whom surgery was achieved exceeding 10 mm. These findings are summarized in Table 1.

### 3.3. Findings of the Studies

#### 3.3.1. Tumor Characteristics

In the six included studies [35,36,37,38,39,40], the main characteristics of the tumor in the participating patients were analyzed. These variables include tumor size, number of metastases, location of the primary tumor site, timing and distribution of metastases, lymph node status, preoperative carcinoembryonic antigen (CEA) levels, tumor differentiation, and disease-free intervals. Tumor size was distinguished into two categories, with a dividing line of five centimeters. In the included studies, the majority of patients had tumors smaller than five centimeters. Regarding the number and type of metastases, there was a relatively balanced distribution of solitary vs. multiple metastases and synchronous vs. metachronous metastases. As the primary location of the tumor, it seems that the colon prevails with almost twice as many reports as the rectum. Positive lymph node status appears to be more common than negative status, and moderately differentiated tumors are the most frequently reported. The distribution of CEA levels shows considerable variability, especially in studies reporting on a larger number of patients. Finally, in the majority of cases, a disease-free interval was observed that did not exceed one year. In these results, Sadot et al. [38] contributed the most substantial patient data for several categories. These tumor characteristics are summarized in Table 2.

#### 3.3.2. Hepatectomy Characteristics

Major hepatectomy, defined as the resection of more than three liver segments, was predominantly performed, a decision that depends on clinical, anatomical, and patient-specific factors. Moreover, in the vast majority of patients, anatomical removal was chosen, i.e., removal of segments, lobes, or subsegments. These data are shown in Table 3.

#### 3.3.3. Neoadjuvant Chemotherapy

In four of the six included studies [37,38,39,40], preoperative chemotherapy was used as indicated by the guidelines. The two chronologically oldest studies [35,36] were conducted before this modification of the guidelines. The use of this preoperative chemotherapy significantly modifies the overall survival of the patients and potentially significantly affects the securing of negative surgical margins by reducing tumor size and addressing microscopic disease. The chemotherapy regimens used in these studies were 5-fluorouracil, oxaliplatin, or irinotecan-based regimens. These data are summarized in Table 4.

#### 3.3.4. Overall Survival

In four included studies [35,36,37,38], the 5-year overall survival of the two groups of surgical margins (1–10 mm and >10 mm) is also reported. The 5-year overall survival rates show a clear prevalence of wider surgical margins in patient survival. In fact, in Kuo et al. [37], surgical margins > 10 mm resulted in greater than twice the 5-year overall survival. Only two studies also reported 5-year disease-free survival [36,37], in which negative surgical margins exceeding 10 mm are also superior. These results suggest that securing wider negative surgical margins may be associated with longer patient survival and lower rates of tumor recurrence. The overall survival rates as well as disease-free rates are depicted in Table 5.

### 3.4. Quality Assessment

**Figure 2:**
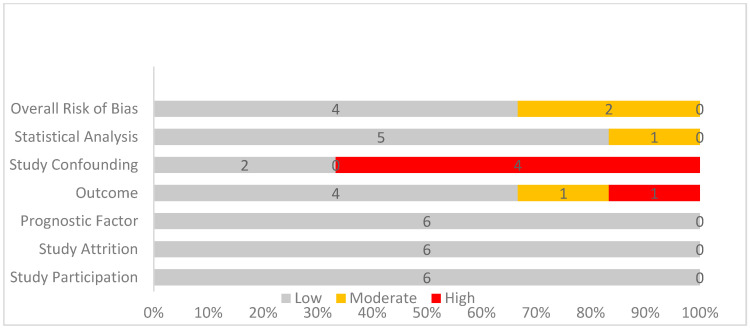
Quality assessment of included studies using the QUIPS tool: bar graph depicting risk of bias levels, with gray indicating low risk, yellow representing moderate risk, and red signifying high risk of bias across evaluated domains.

The QUIPS tool was used to analyze the risk of bias. In the first three of these categories, all studies scored a low risk. However, in the following two categories concerning the risk in outcome measurement and study confounding, one moderate risk of bias and five high risks of bias were noted. Finally, all studies apart from Hughes et al. [35] scored a low risk in the last category regarding statistical analysis and reporting of the results. These results are depicted in Table 6 and Figure 2.

### 3.5. Meta-Analysis Results

#### 3.5.1. Comparison Between Surgical Margins of 1–10 mm and Those Exceeding 10 mm

The meta-analysis included six studies [35,36,37,38,39,40], each reporting HR and 95% CI comparing the survival outcomes of patients with margins greater than 10 mm to those with 1–10 mm negative surgical margins. The overall pooled HR from the random-effects model was 1.38 [1.10; 1.73], indicating that patients with margins between 1 mm and 10 mm had a 38% higher hazard of poorer survival outcomes compared to those with margins exceeding 10 mm. This result was statistically significant, as the CI did not cross 1, suggesting that larger margins may be associated with a better prognosis. Heterogeneity among the studies was moderate, with an I^2^ value of 56%, indicating that about 56% of the variability in effect estimates was due to differences between studies, rather than chance. The τ^2^ value of 0.0408 further quantified the between-study variance.

When conducting the leave-one-out sensitivity analysis, when the study by Sadot et al. [38] was excluded from the analysis, both the heterogeneity and τ^2^ dropped to 0, indicating that the variation between studies was eliminated. The exclusion of the Sadot et al. study also led to a pooled HR of 1.52 [1.30; 1.78], meaning that patients with surgical margins of 1–10 mm were now shown to have a 52% higher hazard of poorer survival. Furthermore, the prediction interval narrowed to [1.18; 1.96], suggesting that future studies are also likely to find a statistically significant harmful effect of larger margins, offering greater certainty about the findings. In contrast, the initial prediction interval of [0.72; 2.63] implied more variability in future studies, potentially leading to non-significant or mixed results.

**Figure 3:**
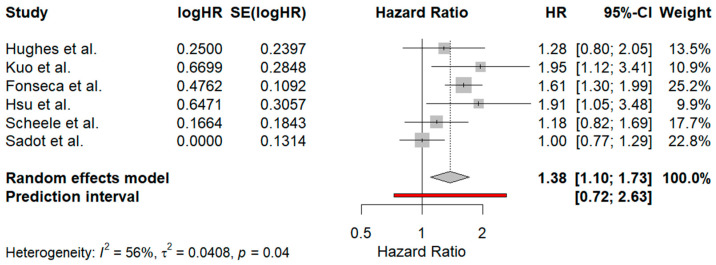
Forest plot summarizing the results of the meta-analysis on the effect of hepatic surgical margins on prognosis in colorectal liver metastases. The pooled hazard ratio (HR) from six studies [35,36,37,38,39,40] indicates that patients with margins between 1 and 10 mm have a 38% higher hazard of poorer survival outcomes compared to those with margins >10 mm (HR: 1.38 [1.10; 1.73]). Moderate heterogeneity is observed (I^2^ = 56%). The red line represents the prediction interval, showing the expected range of true effects in a future study. The dotted vertical line represents the pooled hazard ratio (HR = 1.38) as the central point of the random-effects model estimate.

**Figure 4:**
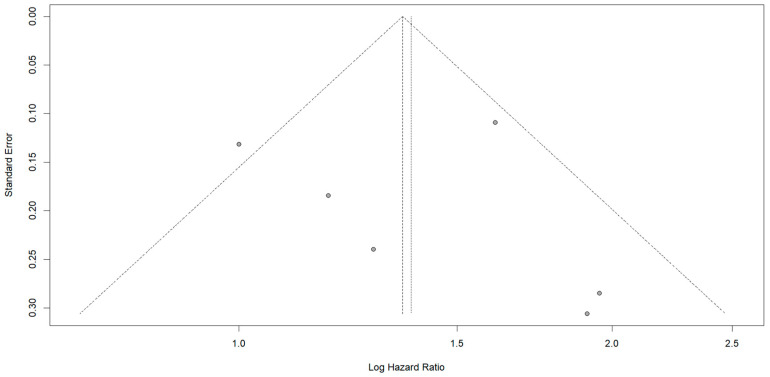
Funnel plot assessing publication bias in the meta-analysis. The slight asymmetry suggests potential publication bias, as smaller studies with non-significant or positive effects of larger margins appear to be underreported or unpublished. The dotted vertical line represents the pooled effect estimate from the meta-analysis.

The funnel plot used to assess publication bias displayed slight asymmetry, with smaller studies missing on one side of the vertical line representing the pooled estimate. This could indicate potential publication bias, where studies showing no significant effect, or a positive effect of larger margins, may have been underreported or not published. The forest plot and the funnel plot are depicted in Figure 3 and Figure 4 respectively.

#### 3.5.2. Subgroup Analysis Based on Preoperative Chemotherapy

**Figure 5:**
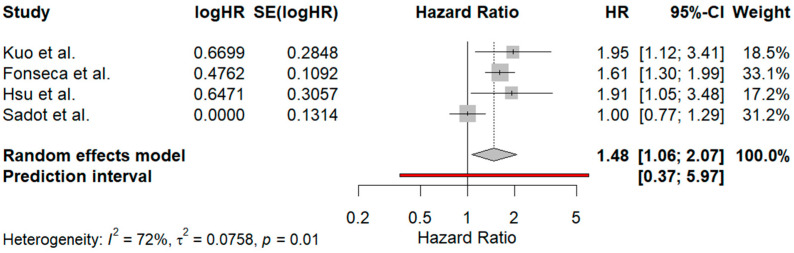
Forest plot from the subgroup analysis assessing the impact of surgical margins greater than 10 mm on the prognosis of patients with colorectal liver metastases (CRLM) in the era of neoadjuvant treatments [37,38,39,40]. The pooled hazard ratio (HR) from four studies indicates a significant survival benefit for patients with margins >10 mm (HR: 1.48 [1.06; 2.07]). The prediction interval of [0.37; 5.97] suggests variability in individual study results, and substantial heterogeneity (I^2^ = 72%, τ^2^ = 0.0758, *p* = 0.01) indicates differences in study designs or populations that may influence the overall effect. The red line represents the prediction interval, showing the expected range of true effects in a future study. The dotted vertical line represents the pooled hazard ratio (HR = 1.48) as the central point of the random-effects model estimate.

A statistical analysis was also performed to investigate the impact of surgical margins greater than 10 mm on the prognosis of patients with CRLM in the era of neoadjuvant treatments [37,38,39,40]. The forest plot presented in Figure 5 demonstrates a random-effects model with a pooled HR of 1.48 [1.06; 2.07], indicating a statistically significant survival benefit for patients with surgical margins greater than 10 mm. The prediction interval of [0.37; 5.97] suggests that while the overall trend leans towards a benefit, individual study results may vary. The heterogeneity across the studies was substantial (I^2^ = 72%, τ^2^ = 0.0758, *p* = 0.01), indicating that differences among the included studies could influence the overall result, possibly due to variations in study design or population characteristics.

The leave-one-out statistical analysis, after excluding Sadot et al. [38], reveals a significant reduction in heterogeneity (I^2^ = 0, τ^2^ = 0), with the *p*-value increasing to 0.74, suggesting a much more consistent effect across the remaining studies. The pooled HR was 1.67 [1.39; 2.02], confirming the survival advantage of the surgical margins greater than 10 mm. The prediction interval of [0.49; 5.72] maintains a broad range.

**Figure 6:**
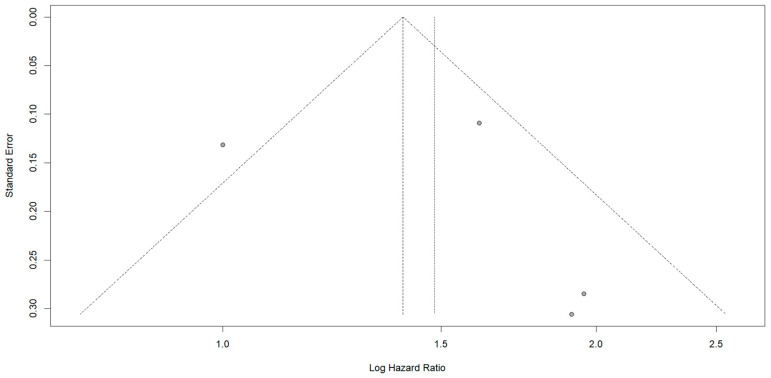
Funnel plot assessing publication bias in the subgroup analysis. The slight asymmetry suggests potential publication bias or small-study effects, indicating that studies with negative or non-significant results may be underreported, potentially influencing the interpretation of the data. The dotted vertical line represents the pooled effect estimate from the meta-analysis.

The funnel plot, as depicted in Figure 6 suggests some asymmetry, which could hint at potential publication bias or small-study effects. This means that smaller studies with negative or non-significant results might be underreported, thus influencing the interpretation of the data.

## 4. Discussion

In this systematic review and meta-analysis, six studies were included [35,36,37,38,39,40], examining the association between surgical margins and patient prognosis in CRLM. Specifically, they analyzed the effect of negative surgical margins at different distances. These distances were 1–10 mm and >10 mm. The analysis revealed that the majority of patients belonged to the 1–10 mm treatment group. Additionally, tumors smaller than 5 cm originating from the colon were prevalent. An even distribution was observed between cases of solitary versus multiple metastases, as well as between synchronous and metachronous metastases. The majority present with positive lymph nodes, indicating the DUKE C stage. The vast majority of tumors were characterized by a moderate differentiation. Anatomical surgery was the primary surgical approach, with major hepatectomies being more common. This selection may aim to preserve liver function, minimize complications, enhance resection margin assessment, and can also be influenced by factors such as the surgeon’s expertise, among others

The results of the meta-analysis were intended to investigate and specifically to compare the effect of negative surgical margins of 1–10 mm with those exceeding 10 mm. The results revealed the significant benefit associated with wider margins. Specifically, pooled HR was 1.38 [1.10; 1.73], indicating a 38% higher risk of poorer survival for patients with smaller margins, suggesting that wider margins are associated with better prognosis. Moderate heterogeneity (I^2^ = 56%) was observed, with τ^2^ = 0.0408, highlighting variability between the studies. A sensitivity analysis excluding Sadot et al. showed a reduction in heterogeneity (I^2^ and τ^2^ = 0), and the HR increased to 1.52 [1.30; 1.78]. This exclusion also led to a narrower prediction interval of [1.18–1.96], indicating that future studies are likely to confirm the beneficial effect of larger margins. Initially, the wider prediction interval of [0.72; 2.63] suggested more uncertainty in potential outcomes.

In the meta-analysis carried out in the subgroup with the aim of investigating the effect of surgical margins greater than 10 mm on the survival of patients with CRLM in the context of neoadjuvant treatments, a pooled hazard ratio (HR) of 1.48 [1.06; 2.07] was observed from a random-effects model. This result indicates a significant survival benefit for patients with wider surgical margins. The prediction interval was calculated [0.37; 5.97], a result that suggests that while most studies trend towards a benefit, individual results could still vary. Substantial heterogeneity was observed (I^2^ = 72%, τ^2^ = 0.0758, *p* = 0.01), suggesting that variations between studies could have impacted the findings. In a leave-one-out sensitivity analysis that excluded the study by Sadot et al. [38], both heterogeneity (I^2^ = 0, τ^2^ = 0) and variance were eliminated, with a *p*-value rising to 0.74. The recalculated HR increased to 1.67 [1.39; 2.02], reinforcing the survival benefit for patients with surgical margins exceeding 10 mm, while the prediction interval [0.49; 5.72] remained broad.

Clinically, these results emphasize the importance of aiming for wider surgical margins (greater than 10 mm) during hepatic resections for CRLM. Surgeons should prioritize margin width as a key factor in improving long-term survival, particularly when patients are undergoing neoadjuvant therapies that may increase the likelihood of achieving these wider margins. This result must be taken into account when planning the hepatectomies strategy, as ensuring surgical margins that exceed 10 mm are associated with better long-term outcomes. The evidence supporting the benefit of wider margins may also influence clinical guidelines and surgical protocols, potentially encouraging more aggressive resections when feasible.

However, negative surgical margins in the 1–10 mm range still provide improved overall survival compared to positive surgical margins. Therefore, in cases where surgical margins greater than 10 mm cannot be achieved, such as when the location of the metastasis prevents this due to the proximity to biliary or vascular structures that need to be preserved, achieving a margin of 1–10 mm remains beneficial [20]. Several factors can impact the accuracy of margin assessment, including the surgical technique and the method used for measuring margins [20]. For instance, certain techniques, like clamp transection, may lead to a loss of tumor-free margin due to differences between preoperative imaging and the actual resection [41]. Additionally, tissue ablation can inadvertently affect the true margin measurement, as the tissue is often altered or removed at the transection site. Variability in the standardization of pathological assessments may also contribute to miscalculations, potentially leading to the overestimation of smaller margins and skewing the evaluation of surgical success [20,42,43].

The management of CRLM often presents a significant challenge, with treatment guidelines showing minimal variation across continents [44,45,46]. The therapeutic strategy is primarily adjusted based on the resectability of the metastases [10]. However, at the time of diagnosis of liver metastasis, approximately one in five cases is considered suitable of resection [47,48]. For the majority of cases with initially unresectable tumors, chemotherapy is recommended to achieve resectability, as this resection ensures improved survival rates [47,48]. Another challenge involves the diagnosis of synchronous CRLM, which also occurs in approximately one in five cases. In these cases, simultaneous minimally invasive (MI) surgery represents a safe option, offering advantages such as faster recovery and shorter postoperative hospital stays, as well as reduced rates of intraoperative and postoperative complications [49,50]. However, simultaneous resection is typically reserved for low-risk patients, as high-risk patients face elevated mortality rates [51]. Amid of multiple bilobar CRLM, the liver-first approach is the best approach as it is associated with improved outcomes [52]. Therefore, resectable tumors can be treated with three types of approaches. These are upfront liver surgery followed by adjuvant chemotherapy, perioperative chemotherapy preceding and following liver surgery, and an upfront systemic treatment including chemotherapy plus a targeted agent, both chosen according to patients’ and tumors’ characteristics, then followed by liver surgery if indicated [53]. The choice of each approach is based on the clinical, radiological, pathological, and molecular features of the tumor and the patient. Finally, palliative systemic therapy is used for unresectable tumors. In these patients, liver transplantation is being studied extensively to establish a new therapeutic approach [52].

Numerous factors influence the OS of patients undergoing hepatectomy. Many of these factors relate to patient demographics or tumor characteristics. Specifically, some of the characteristics of the tumor that affect DFS or OS are the synchronous or metachronous presentation of the tumor, the T stage, the involvement or not of the lymph nodes, and the number of liver lesions. The patients with a synchronous appearance, with stage T4, with lymph node involvement as well as the multiple number of liver lesions have a worse prognosis [54,55,56]. Regarding the number of CRLMs, more than four significantly affect the prognosis [57]. In addition, the presence of micrometastases significantly affects the prognosis of patients and is associated with the presence of the RAS/TP53 mutation [58]. A mass size greater than 4 cm is another factor associated with a worse prognosis [59]. Finally, complications of surgical treatment are prognostic indicators, such as surgical site infection [60]. The localization of the tumor does not significantly affect the prognosis [61]; however, the surgical margins affect to a greater extent the left-sided primary metastasis [62]. The prognosis of patients is also not associated with anti-platelet therapy [63].

The technique and type of surgery also play a significant role in influencing the prognosis of patients with CRLM. Regarding the type of operation, it seems that both the laparoscopic technique and the open surgery are appropriate, since they are accompanied by similar OS and DFS rates [64]. However, the laparoscopic technique is accompanied by better surgical margins, a shorter length of stay, and less morbidity [65]. Yet, not all patients are suitable for laparoscopic surgery. Thus, characteristics such as liver function and tumor size must be taken into account before choosing surgery [66,67]. The use of virtual reality can also improve the performance of the laparoscopic technique [68]. Finally, robot-assisted liver surgery presents some advantages, such as reduced morbidity and mortality [69,70]. Regarding the surgical technique, both anatomical and non-anatomical resections are safe and feasible techniques. Non-anatomical resections are accompanied by less surgical time and less blood transfusion [71,72]. Also, the time to surgery seems to affect the prognosis, with short and intermediate times being accompanied by a higher OS [73].

Additionally, the prognosis of patients also hinges on the re-resection. CRLM is a metastatic disease with relatively high recurrence rates [74]. In these patients, repeat hepatectomy can be utilized as a potential salvage therapy. Specifically, repeat hepatectomy is accompanied by low rates of morbidity and mortality and offers the possibility of longer survival for patients with CRLM [75,76,77,78]. However, the success of the repeat hepatectomy also depends on many factors, just like the initial hepatectomy. These include high CEA levels, multiple tumors, disease-free intervals, less time, large diameter tumors, as well as positive surgical margins [79].

The presence of numerous prognostic factors has underscored the necessity for a prognostic index. Thus, many prognostic scores have been proposed. Characteristics such as tumor size, the number of metastases, classification according to TNM classification, DFI, CEA levels, major or minor resection, and other preoperative and surgical factors appear to be prominent in these scores [80,81,82]. Surgical margins are also prominent in these scores. The main feature is the presence of positive or negative surgical margins; however, the exact distance differs between the scores, with some suggesting 1 mm [82,83], others 5 mm [80,84], and others 10 mm. This dichotomy also highlights the need for a direct comparison of surgical margins, as accomplished by this systematic review and meta-analysis.

Several risk factors are associated with positive surgical margins in patients undergoing hepatectomies. Specifically, these factors include the type of operation, the number of metastases, their size, and distribution across lobes. Thus, in patients with large masses, multiple metastases, and more widespread distribution, there is an increased risk for positive surgical margins. Furthermore, the non-desmoplastic growth pattern is associated with an increased risk of positive surgical margins. In addition, in patients undergoing laparoscopic operations, blood loss is another risk factor. These factors make it difficult to ensure negative surgical margins, necessitating the existence of techniques that can contribute to the expansion of surgical margins [85,86,87].

Another factor that significantly influences both the risk of local recurrence and the success of negative surgical margins is the KRAS status [88]. Specifically, in patients with KRAS metastases, there is an increased risk of positive surgical margins and narrower margins [89]. Also in these patients, there is an increased risk of local recurrence, which is less prevalent among patients who achieved negative surgical margins as in those with positive surgical margins and wild-type KRAS (wtKRAS) [90]. Therefore, in wtKRAS, to avoid this high risk of recurrence, it is suggested to ensure at least 5 mm negative surgical margins and not 1 mm [91,92].

Numerous techniques have been studied in an effort to increase the extent of healthy surgical margins. The use of indocyanine green fluorescence imaging is one of the techniques, which significantly contributes to ensuring negative surgical margins. It is an easy and accessible intraoperative imaging technique for liver surgery. This technique gives real-time surgical margin assessment, especially during minimally invasive and robotic surgeries. Thus, it allows to ensure negative surgical margins >1 mm [93,94,95,96]. In addition, contrast-enhanced intraoperative ultrasound and label-free multimodal multiphoton microscopy contribute to ensuring surgical margins [97,98].

There are additional systematic reviews and meta-analyses in the available literature that investigate the influence of surgical margins on overall survival following hepatic resection for colorectal metastases, aside from the present study [21,99,100]. However, this meta-analysis takes a novel approach by directly comparing the two most commonly selected thresholds for negative surgical margins (1–10 mm and >10 mm) and their impact on survival. In contrast, previously published meta-analyses focus on comparing negative margins to positive ones, using either 1 cm as the cut-off for defining negativity or grouping 1 mm with >1 cm, without specifically isolating the 1–10 mm range. Furthermore, two of the three [99,100] available meta-analyses rely on relative risks or odds ratios to assess outcomes, which do not account for the time parameter. In contrast, this study utilizes hazard ratios (HRs), offering a more time-sensitive and comprehensive measure of the outcome.

The findings of this systematic review and meta-analysis hold significant implications for clinical practice. Highlighting negative surgical margins as a crucial prognostic indicator, the study underscores that securing negative surgical margins exceeding 10 mm is correlated with markedly higher rates of patient survival. Nonetheless, several factors may impede the attainment of desired surgical margins. In summary, it is imperative in clinical practice to strive for negative surgical margins exceeding 1 mm, with the overarching aim of maximizing margin width whenever possible.

This study has some limitations, the main one being heterogeneity. A great difference is observed between the studies, regarding both the recruitment period and the surgical techniques chosen. Still, the lack of information in the studies regarding the histopathological assessment, the experience of the surgeons, and the time that elapsed from the surgery to the histopathological assessment are limitations. In addition, the heterogeneity between the number of participants in each study, as well as in each group, is another limitation. Also, the presence of many prognostic factors, concerning both tumor characteristics and patient demographics, may affect the prognostic results and is a limitation.

1. Restrospective studies only.
2. Data from other reports, with inadequate information reporting, could not be included in the present synthesis.

Finally, the lack of randomized studies in the literature limits the reliability of the results and does not allow the validation of a causative effect. Given the limitations inherent to the retrospective nature of the included studies and potential biases associated with data collection and reporting, prospective studies with standardized protocols and long-term follow-ups are necessary to confirm and expand upon our findings.

Therefore, randomized studies are required, which directly compare these surgical margins and ideally eliminate the influence of other prognostic factors through proper patient allocation. Also, it is worth carrying out further investigations with subgroups in these surgical margins, to investigate factors that directly affect the prognosis in combination with the surgical resection margin. Further investigations can also be carried out on the results of surgical margins in long-term follow-up studies. Furthermore, investigating the role of adjuvant therapies, such as chemotherapy or targeted therapy, in conjunction with surgery and its impact on outcomes based on different margin widths is necessary. Finally, it is useful to carry out further investigations concerning the influence of the surgical limits on the quality of life or even on the cost-effectiveness of each technique.

## 5. Conclusions

In conclusion, the findings of this systematic review and meta-analysis suggest that larger surgical margins (>10 mm) are associated with better overall survival compared to smaller margins (1–10 mm). Subgroup analysis further confirmed these findings in the era of neoadjuvant treatments, with an even higher HR. These findings provide insights into the importance of surgical margin width in predicting survival outcomes for patients with colorectal liver metastases. Clinically, this suggests that surgeons should aim for margins greater than 10 mm whenever feasible to improve patient prognosis.

## Figures and Tables

**Table 1:** Characteristics of the included studies. R0 refers to the negative surgical margin and R1 to the positive surgical margin. Abbreviations: ORCS: observational retrospective cohort study.

Study ID	Location	Type of Research	Total Subjects (%Male/Age)	Recruitment Period	Substratification of Margins (n)	Substratification of Negative Margins (n)	Median Follow-Up (Months)
Hughes et al., 1988 [35]	California, US	ORCS	100 -	1948–1985	R0 (26) R1 (1)	1–10 mm: 9 >10 mm: 17	≈60
Scheele et al., 1995 [36]	Erlanger, Germany	ORCS	434 (52.8/59)	1960–1992	R0 (350) R1 (48)	1–10 mm: 204 >10 mm: 146	>60
Kuo et al., 2015 [37]	Taoyuan, Taiwan	ORCS	159 (55.9/-)	1991–2006	-	1–10 mm: 128 >10 mm: 31	38.5
Sadot et al., 2015 [38]	New York, US	ORCS	2368 (57/61)	1992–2012	-	1–10 mm: 1191 >10 mm: 765	55
Fonseca et al., 2022 [39]	Sao Paulo, Brazil	ORCS	101 (54.5/63)	2007–2015	-	1–10 mm: 66 >10 mm: 35	34
Hsu et al., 2024 [40]	Taoyuan, Taiwan	ORCS	230 (63.5/62.4)	2010–2019	R0 (224) R1 (4)	1–10 mm: 166 >10 mm: 62	39

**Table 2:** Tumor characteristics.

Study ID	Tumor Size (mm)		No. of Metastases		Location of the Primary Site		Metastases		Distribution for Multiple Metastases		Status of Primary Tumor Lymph Nodes		Preoperative CEA Level		Differentiation			Disease-Free Interval	
	<5 cm	>5 cm	Solitary	Multiple	Colon	Rectum	Synchronous	Metachronous	Unilobar	Bilobar	Negative	Positive	<	>	W	M	P	<1 Year	>1 Year
Hughes et al., 1988 [35]	-	-	81	16	-	-	-	-	13	3	41	30	-	-	-	-	-	64	34
Scheele et al., 1995 [36]	233	117	203	147	189	161	142	208	90	57	116	216	82 <5 ng/mL	197 >5 ng/mL	-	-	-	77	111
Kuo et al., 2015 [37]	129	30	101	58	63	69	104	55	114	45	32	127	-	-	15	138	6	-	-
Sadot et al., 2015 [38]	1654	714	1010	1358	-	-	1181	1187	-	-	907	1440	1950 <200 μg/L	178 ≥200 μg/L	-	-	-	1504	864
Fonseca et al., 2022 [39]	80	21	-	-	57	44	62	39	-	-	47	45	65 <200 μg/L	32 ≥200 μg/L	-	-	-	-	-
Hsu et al., 2024 [40]	-	-	230	0	230	0	121	109	-	-	146	82	97 <5 ng/mL	131 >5 ng/mL	12	203	14	-	-
Total	2096	882	1625	1579	539	274	1610	1598	217	105	1289	1940	-	-	27	241	20	1645	1009

**Table 3:** Hepatectomy characteristics.

Study ID	Major Hepatectomy	Minor Hepatectomy	Anatomical	Nonanatomical
Hughes et al., 1988 [35]	47	44	-	-
Scheele et al., 1995 [36]	-	-	291	59
Kuo et al., 2015 [37]	-	-	32	127
Sadot et al., 2015 [38]	1215	871	-	-
Fonseca et al., 2022 [39]	36	65	-	-
Hsu et al., 2024 [40]	-	-	-	-
Total	1298	980	323	186

**Table 4:** Neoadjuvant chemotherapy.

Study ID	Number of Patients	Regimen	5-Year Overall Survival (%)	
			Yes	No
Hughes et al., 1988 [35]	-	-	-	-
Scheele et al., 1995 [36]	-	-	-	-
Kuo et al., 2015 [37]	15 (9.4%)	5-fluorouracil-based	35.7	20
Sadot et al., 2015 [38]	2193 (93%)	Oxaliplatin or irinotecan	43	36
Fonseca et al., 2022 [39]	101 (100%)	Oxaliplatin	-	-
Hsu et al., 2024 [40]	-	-	-	-

**Table 5:** Overall survival. The asterisk (*) indicates that the percentage is actual and not actuarial.

Study ID	5-Year Overall Survival (%)		5-Year Disease-Free (%)	
	1–10 mm	>10 mm	1–10 mm	>10 mm
Hughes et al., 1988 [35]	20 *	30 *	-	-
Scheele et al., 1995 [36]	37	43	31	37
Kuo et al., 2015 [37]	18.2	40.2	28.4	58.1
Sadot et al., 2015 [38]	46	48	-	-
Fonseca et al., 2022 [39]	-	-	-	-
Hsu et al., 2024 [40]	-	-	-	-

**Table 6:** Quality assessment of included studies. The QUIPS tool was utilized. The positive sign (green) symbolizes low risk, the negative sign (yellow) moderate, while the X symbol (red) indicates high risk of bias.

Study ID	Study Participation	Study Attrition	Prognostic Factor Measurement	Outcome Measurement	Study Confounding	Statistical Analysis and Reporting	Overall Risk of Bias
Hughes et al., 1988 [35]	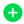	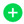	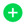	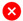	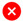	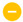	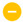
Scheele et al., 1995 [36]	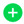	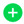	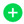	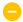	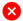	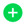	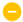
Kuo et al., 2015 [37]	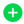	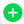	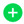	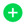	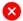	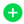	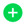
Sadot et al., 2015 [38]	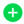	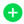	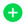	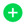	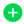	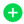	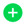
Fonseca et al., 2022 [39]	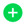	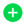	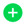	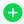	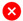	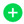	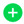
Hsu et al., 2024 [40]	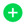	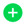	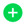	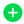	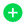	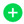	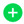

## Data Availability

No new data were created in this study. Data sharing is not applicable to this article.
